# Supplementary material for: EMT activates ER-to-Golgi trafficking through upregulation of REEP2 to promote lung cancer progression
Source: Res Sq. 2026 Jul 22:rs.3.rs-8117355. Preprint. [Version 1] doi: 10.21203/rs.3.rs-8117355/v1 (PMC13419587; doi:10.21203/rs.3.rs-8117355/v1)
Supplement: 1 [file NIHPPRS8117355V1-supplement-1.pdf]

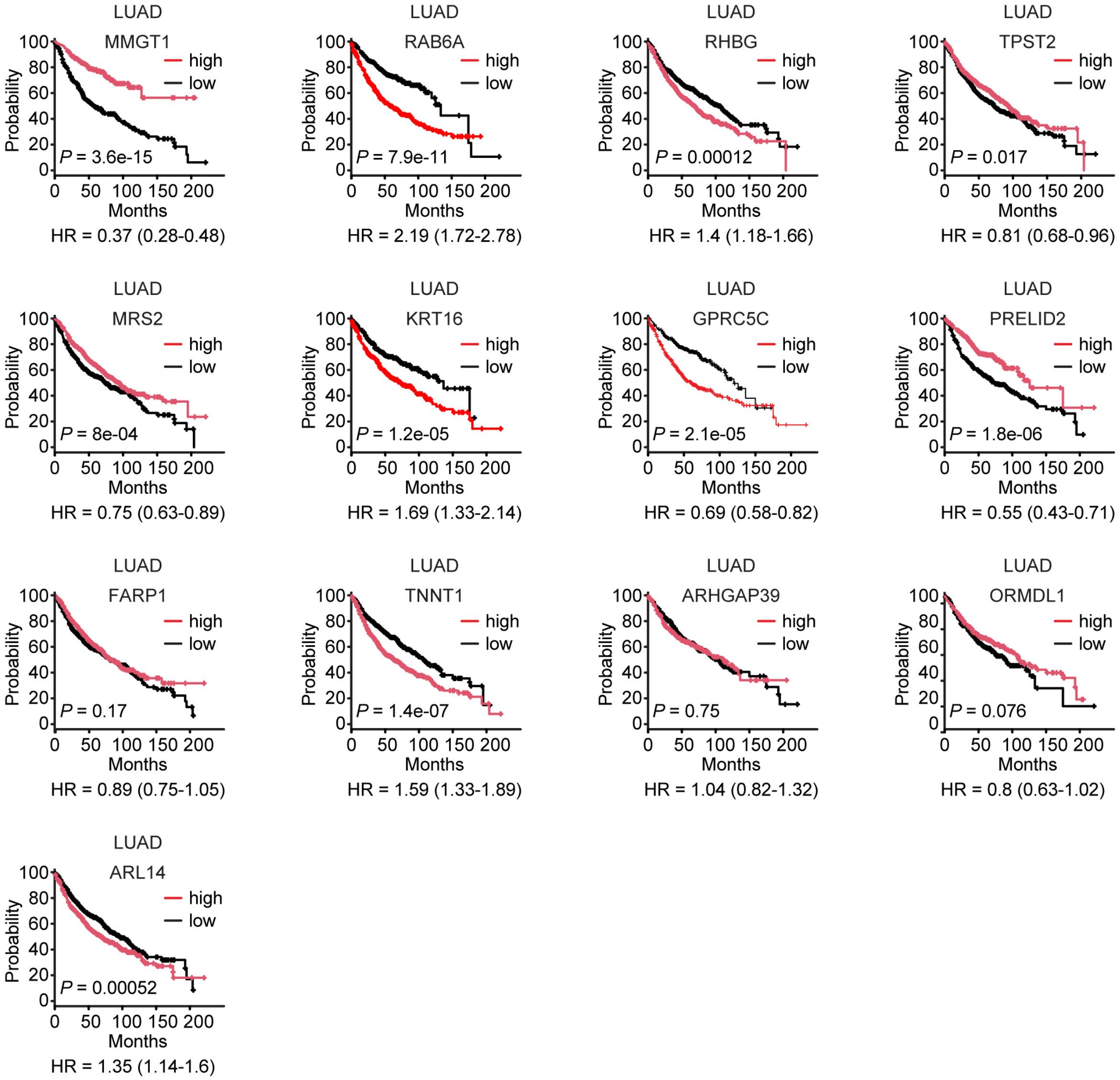

Fig. S1. Kaplan-Meier survival analysis of LUAD patients based on the mRNA levels of indicated genes above (high) or below (low) the median value.

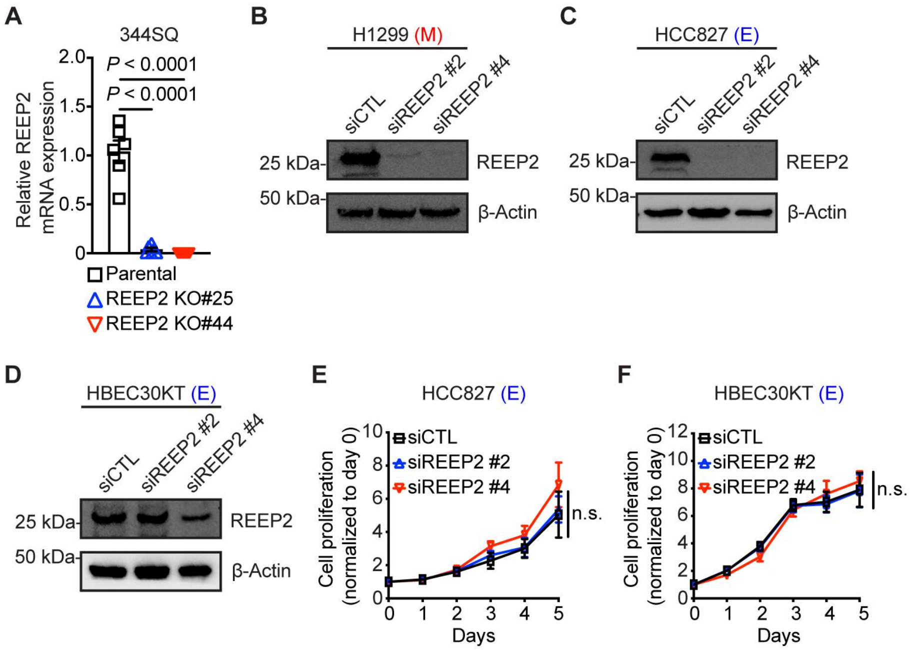

Figure S2. Depletion of REEP2 in murine and human LUAD cells. (A) qPCR analysis of REEP2 mRNA expression levels in parental or REEP2 knockout (KO) 344SQ cell lines (n=6 replicates per condition). (B-D) WB analysis of REEP2 levels in human H1299 (B), HCC827 (C), and HBEC30KT (D) cells transfected with control siRNA (siCTL) or REEP2 siRNA (siREEP2). β-Actin loading control. M: mesenchymal. E: epithelial. (E, F) *In vitro* cell proliferation assay in human HCC827 (E) and HBEC30KT (F) cells transfected with control siRNA (siCTL) or REEP2 siRNA (siREEP2). Results represent means ± SEM. *P* values were determined using two-tailed Student’s t-test.

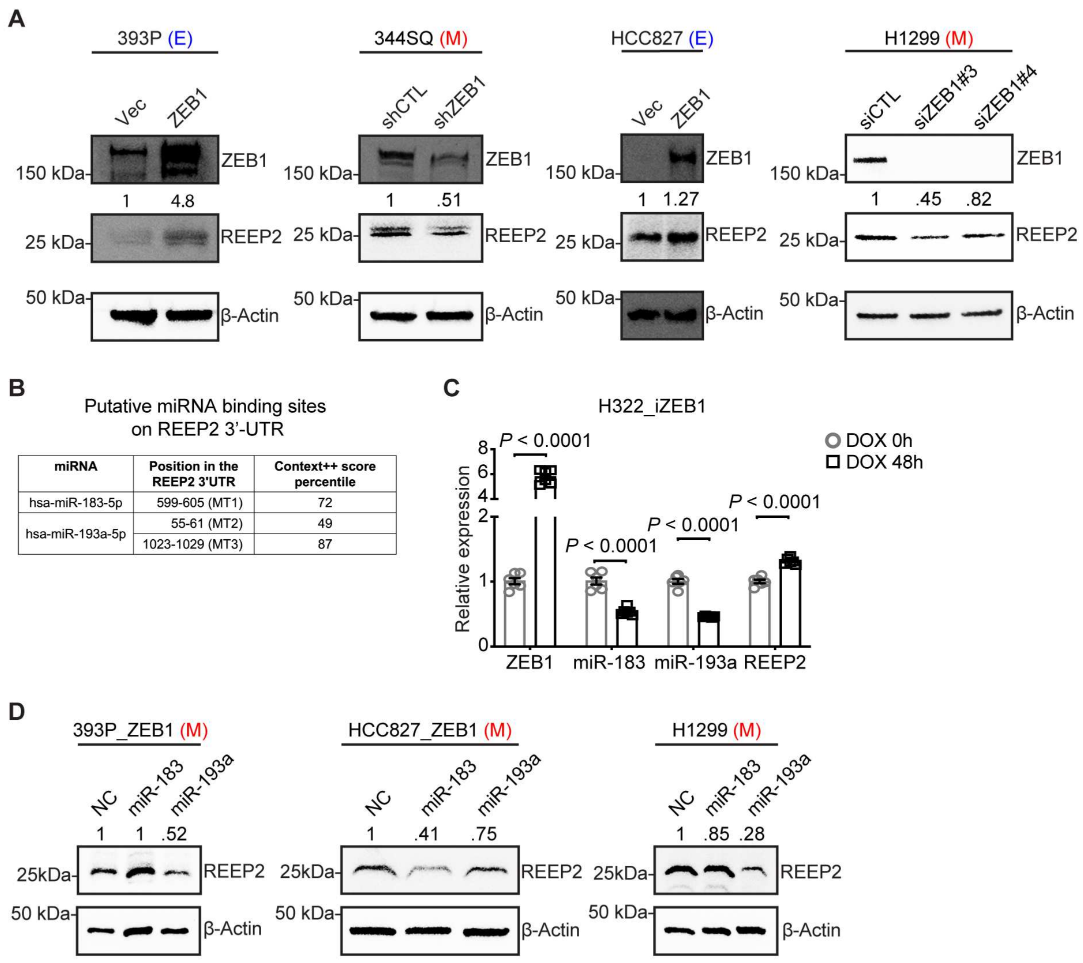

miRNA	Position in the REEP2 3'UTR	Context++ score percentile
hsa-miR-183-5p	599-605 (MT1)	72
hsa-miR-193a-5p	55-61 (MT2)	49
	1023-1029 (MT3)	87

Figure S3. ZEB1 upregulates REEP2 through the regulation of miRNAs. (A) WB analysis of ZEB1 and REEP2 protein expression levels in murine (393P, 344SQ) and human (HCC827, H1299) LUAD cells with ectopic ZEB1-expression or ZEB1-depletion. β-actin loading control. Relative densitometric values are indicated. E: epithelial. M: mesenchymal. (B) Putative miRNA binding sites on REEP2 3'UTR predicted by TargetScan and used for mutagenesis (MT1-3) (C) qPCR analysis of ZEB1, miR-183, miR-193a, and REEP2 expression levels in human H322_iZEB1 cells with doxycycline (DOX) (1 μg/ml) treatment (n=6 replicates per condition). Results represent means ± SEM. *P* values were determined using two-tailed Student's t-test. (D) WB analysis of REEP2 protein expression levels in the indicated cells transfected with miR-183 or miR-193a mimics or non-coding control (NC). M: mesenchymal.

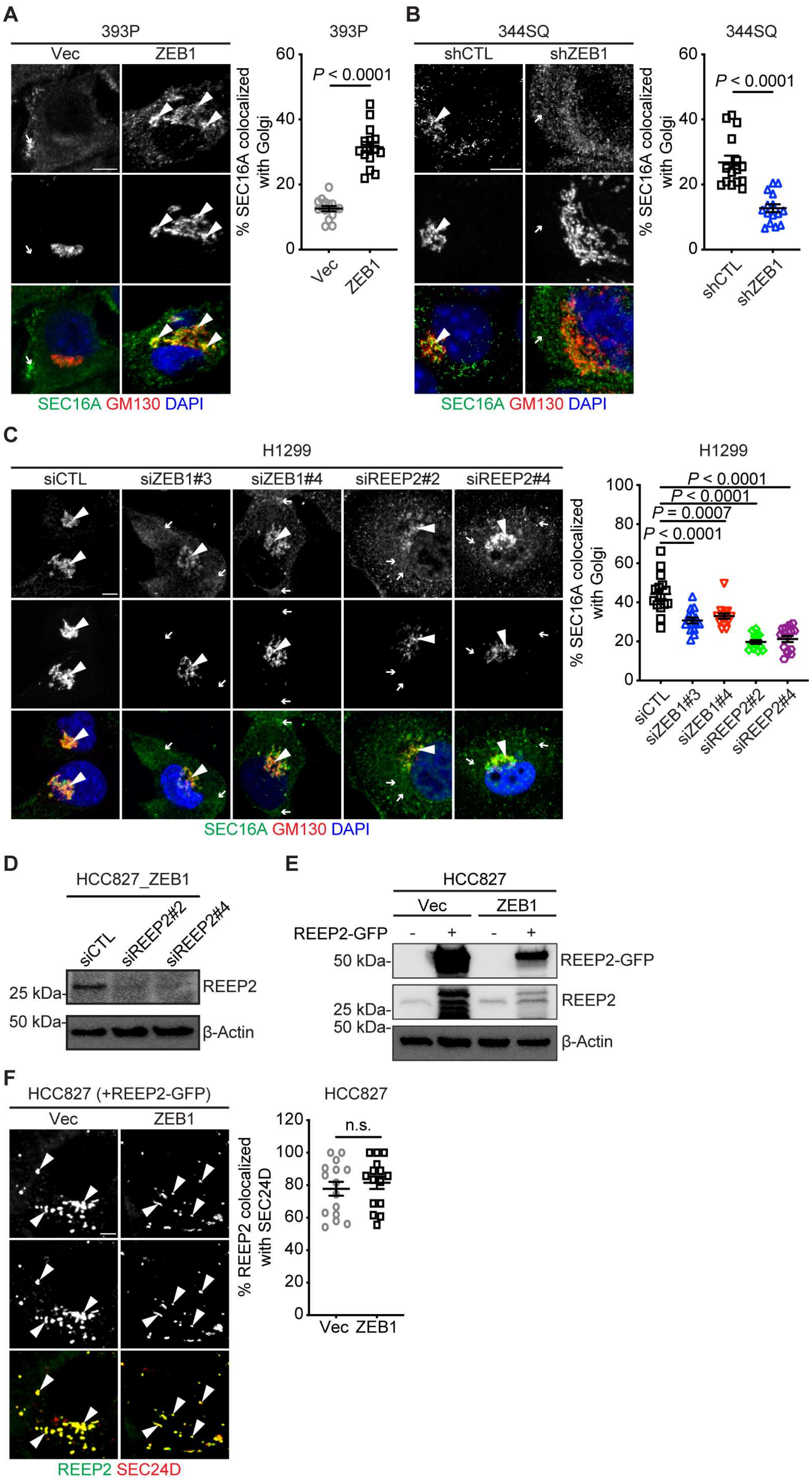
A
393P
Vec
ZEBI
SEC16A GM130 DAPI
% SEC16A colocalized with Golgi
P < 0.0001
60
40
20
0
Vec
ZEB1
B
344SQ
shCTL
shZEB1
SEC16A GM130 DAPI
P < 0.0001
shCTL
shZEB1
C
H1299
siCTL
siZEB1#3
siZEB1#4
siREEP2#2
siREEP2#4
SEC16A GM130 DAPI
P < 0.0001
P < 0.0001
P = 0.0007
P < 0.0001
100
80
D
HCC827_ZEB1
siCTL
siREEP2#2
siREEP2#4
25 kDa-
50 kDa-
REEP2
β-Actin
E
HCC827
Vec
ZEB1
REEP2-GFP
50 kDa-
25 kDa-
50 kDa-
REEP2-GFP
REEP2
β-Actin
F
HCC827 (+REEP2-GFP)
Vec
ZEB1
REEP2 SEC24D
HCC827
% REEP2 colocalized with SEC24D
n.s.
120
100

Figure S4. ZEB1 drives ERES colocalization with the Golgi via REEP2. (A-C) Confocal micrographs of cells co-stained with anti-SEC16A (green) and anti-GM130 (red) antibodies. DAPI (blue). Scale bar: 5 μm. The scatter plots quantify Golgi-localized SEC16A per cell (dot) based on % of total SEC16A that co-localizes with Golgi (GM130 channel) (n = 15 cells per group) in murine epithelial cells (393P) with ectopic ZEB1-expression (A), in murine mesenchymal cells (344SQ) with ZEB1 depletion (B), or in human mesenchymal cells (H1299) with ZEB1 or REEP2 depletions (C). (D) WB analysis of REEP2 protein expression levels in HCC827_ZEB1 cells transfected with control siRNA (siCTL) or REEP2 siRNA (siREEP2). β-actin loading control. (E) WB analysis of REEP2 protein expression levels in HCC827 cells co-transfected with empty vector (Vec) or ZEB1, with REEP2-GFP. β-actin loading control. (F) Confocal micrographs of cells co-transfected with REEP2-GFP (green) and SEC24D-mCherry (red). Scale bar: 5 μm. The scatter plots quantify SEC24D-localized REEP2 per cell (dot) based on % of total REEP2 that co-localizes with SEC24D (n = 15 cells per group) in HCC827 cells with ectopic ZEB1-expression. Results represent means ± SEM. *P* values were determined using two-tailed Student's t-test.

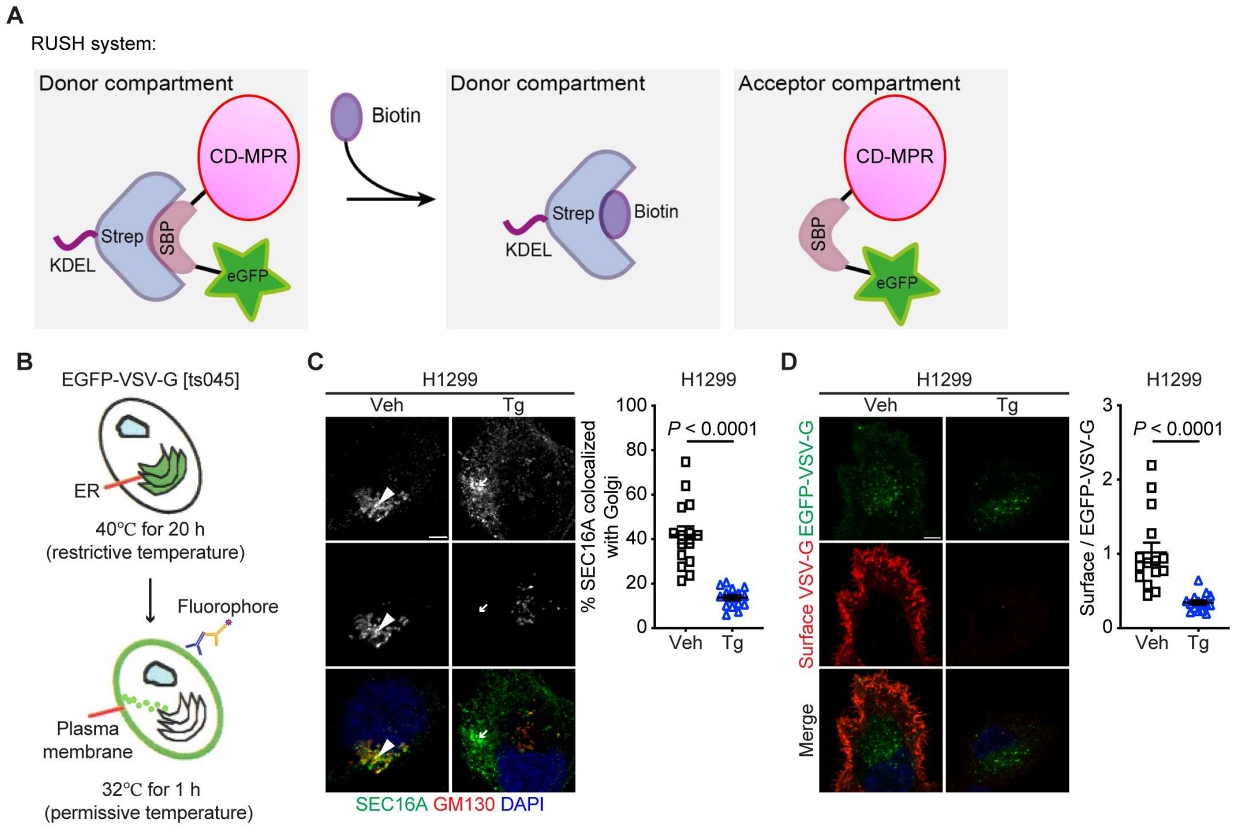

Figure S5. ERES/Golgi colocalization promotes secretory trafficking. (A) Schematic illustration of the retention using selective hooks (RUSH) system. The cells were co-transfected with the reporter protein (CD-MPR), which is fused to the streptavidin-binding peptide (SBP) and GFP, and a second protein with a C-terminal ER retention signal (Lys-Asp-Glu-Leu; KDEL) fusing with streptavidin as a hook. The interaction of CD-MPR with the hook protein retains CD-MPR in the ER compartment and biotin administration releases the CD-MPR-GFP reporter from the ER and transfers it to the Golgi. (B) Schematic of VSV-G assay. The cells were transfected with vectors that express an EGFP-tagged temperature-sensitive mutant VSV-G (EGFP-VSV-G [ts045]) that is transported from the ER to the plasma membrane via the Golgi. At designated time points after switching cells to temperatures that cause VSV-G accumulation (40°C) and release (32°C) from the ER, cells were fixed and exofacial VSV-G was detected in non-permeabilized cells by staining with anti-VSV-G monoclonal antibody. The VSV-G trafficking to the plasma membrane is determined by the ratio of exofacial (surface) VSV-G fluorescence signal to the

EGFP signal intensity. (C) Confocal micrographs of cells co-stained with anti-SEC16A (green) and anti-GM130 (red) antibodies. DAPI (blue). Scale bar: 5 µm. The scatter plots quantify Golgi-localized SEC16A per cell (dot) based on % of total SEC16A that co-localizes with Golgi (GM130 channel) (n = 15 cells per group) in H1299 cells treated with vehicle control (Veh) or 5 µM thapsigargin (Tg) for 1 h. (D) Confocal micrographs of EGFP-VSV-G-transfected cells taken 1 h after transfer to permissive temperature. Scale bar, 5 µm. The scatter plot represents the ratio of surface VSV-G to EGFP-VSV-G in each cell (dot) (n = 15 cells per group) in H1299 cells treated with vehicle control (Veh) or 5 µM thapsigargin (Tg) for 1 h. Results represent means ± SEM. *P* values were determined using two-tailed Student's t-test.

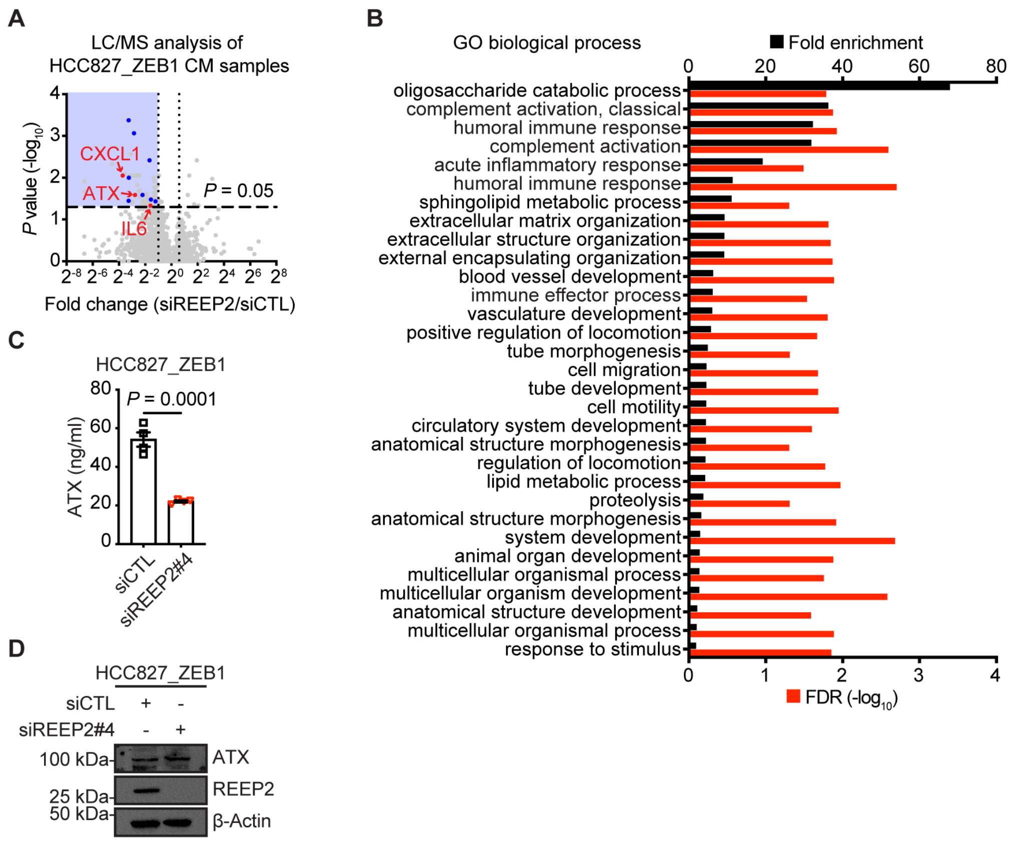

Figure S6. REEP2 activates a pro-metastatic secretion. (A) Volcano plot of proteins identified by LC/MS analysis of CM samples. *P* values (Y axis) and fold-change (X axis). Blue box indicates proteins with significantly reduced secretion (blue box, $P < 0.05$) in REEP2-depleted HCC827_ZEB1 cells (siREEP2). The blue and red dots label the factors that are associated with immune regulation. (B) Enrichment analysis (Fisher's exact test using Gene Ontology terms) of the down-regulated secreted proteins. (C) ELISA assay shows the ATX levels in CM samples (n = 4 replicates per group). *P* values were determined using two-tailed Student's t-test. (D) WB analysis of ATX and REEP2 levels in human HCC827_ZEB1 cells transfected with control siRNA (siCTL) or REEP2 siRNA (siREEP2#4). β-actin loading control.
